# Cellular Microvesicle Pathways Can Be Targeted to Transfer Genetic Information between Non-Immune Cells

**DOI:** 10.1371/journal.pone.0006219

**Published:** 2009-07-13

**Authors:** Amy M. Skinner, S. Lee O'Neill, Peter Kurre

**Affiliations:** 1 Papé Family Pediatric Research Institute, Oregon Health & Science University, Portland, Oregon, United States of America; 2 Departments of Pediatrics, Oregon Health & Science University, Portland, Oregon, United States of America; 3 Cell & Developmental Biology, Oregon Health & Science University, Portland, Oregon, United States of America; Universität Heidelberg, Germany

## Abstract

Eukaryotic cell communication is based on protein signaling cascades that require direct cell-cell apposition, or receptor engagement by secreted molecules. The transmission of genetic information is thought to be uncommon, apart from recent reports of exosomal RNA transfer in immune and glioblastoma cells. We wished to examine if existing microvesicle pathways could be directly targeted for the horizontal transfer of RNA genomes in less specialized cell types. Using replication-deficient retrovirus vector, studies herein confirm that a range of cells routinely sequester a small population of these RNA genomes in a non-canonical compartment, refractory to antibody neutralization and unaffected by specific pharmacological inhibition of pathways involved in conventional viral trafficking. Our experiments further reveal the cytoplasmic colocalization of vector genomes with tetraspanin proteins as well as the PI-3-kinase sensitive trafficking and subsequent transmission to 2° targets. Collectively, our results indicate a scalable process whereby cells route vector genomes to multivesicular bodies (MVB) for cytoplasmic trafficking and exosomal release. Our findings imply that cells can serve to deliver recombinant payload, targeted for the stable genetic modification of 2° target cells.

## Introduction

Eukaryotic cell communication is based on protein signaling via direct cell-cell contacts, or indirectly via ligand-receptor interactions. Recent work suggests that cell-cell communication may occur in part through transfer in membrane-derived vesicles that stem from the fusion of multivesicular bodies (MVB) with the plasma membrane [1]. Unlike the exchange of DNA episomes seen in prokaryotes, the cell membrane and cytoplasmic environment in higher order species present a substantial barrier for the trafficking of nucleic acids. The recently described microvesicle transfer of RNA between glioblastoma cells or the exosomal cell-cell transmission of microRNA in mast cells provide highly specialized exceptions of “horizontal” genetic communication among target cells [2], [3]. Fundamentally, those studies demonstrate microvesicle mediated transfer and cytoplasmic detection of donor cell “RNA signatures” in 2° targets. Little is known about the recruitment and trafficking of RNA to such a pathway and its potential existence in less specialized cell populations. Specifically, there have been no demonstrations of long-lived effects in 2° targets, nor attempts to directly exploit such genetic communication.

During recent studies investigating the cell-cell transfer of replication incompetent VSV-G pseudotyped particles, we observed a population of intracellularly captured particles refractory to neutralization by envelope-specific antibody or protease, and capable of 2° transfer [4], [5]. Based on these intriguing observations, we hypothesized that replication deficient RNA vector genomes might be subject to recruitment into a microvesicle transfer pathway. In sharp distinction to prior studies that rely on endogenous protein and RNA cargo, tagged retrovirus vectors allow us for the first time to prospectively follow genome trafficking in the donor (1° target) cell. Late generation HIV-1 derived lentiviral vector particles use split packaging designs and their RNA transfer genome is devoid of open reading frames required for viral replication, collectively intended to prevent mobilization, packaging and spread of the vector genome. Viral replication incompetence truncates the vector life cycle, and conceptually, replication-incompetent retrovirus is thought to follow one of two fates upon cell entry: nuclear translocation and integration at its genomic destination, or rapid cytoplasmic degradation in lysosomes or proteasomes [6], [7]. Replication deficiency therefore avoids bias from viral assembly and trafficking during egress, and provides a sensitive experimental system with readily traceable, stable biologic effects in the 2° target.

We now demonstrate that cells sequester genomes in a non-canonical microvesicle compartment enriched in tetraspanin proteins where they bypass routing to the nucleus, escape a degradative fate and transfer to a 2° target. Cytoplasmic trafficking is susceptible to inhibition of phosphatitdyl inositol-3-kinase (PI-3-K) activity and can be exploited for the deliberate and scalable cellular delivery of integrating genetic sequence.

## Results

### Cells retain recombinant vector genomes in a protease-resistant intracellular compartment

**Figure 1:**
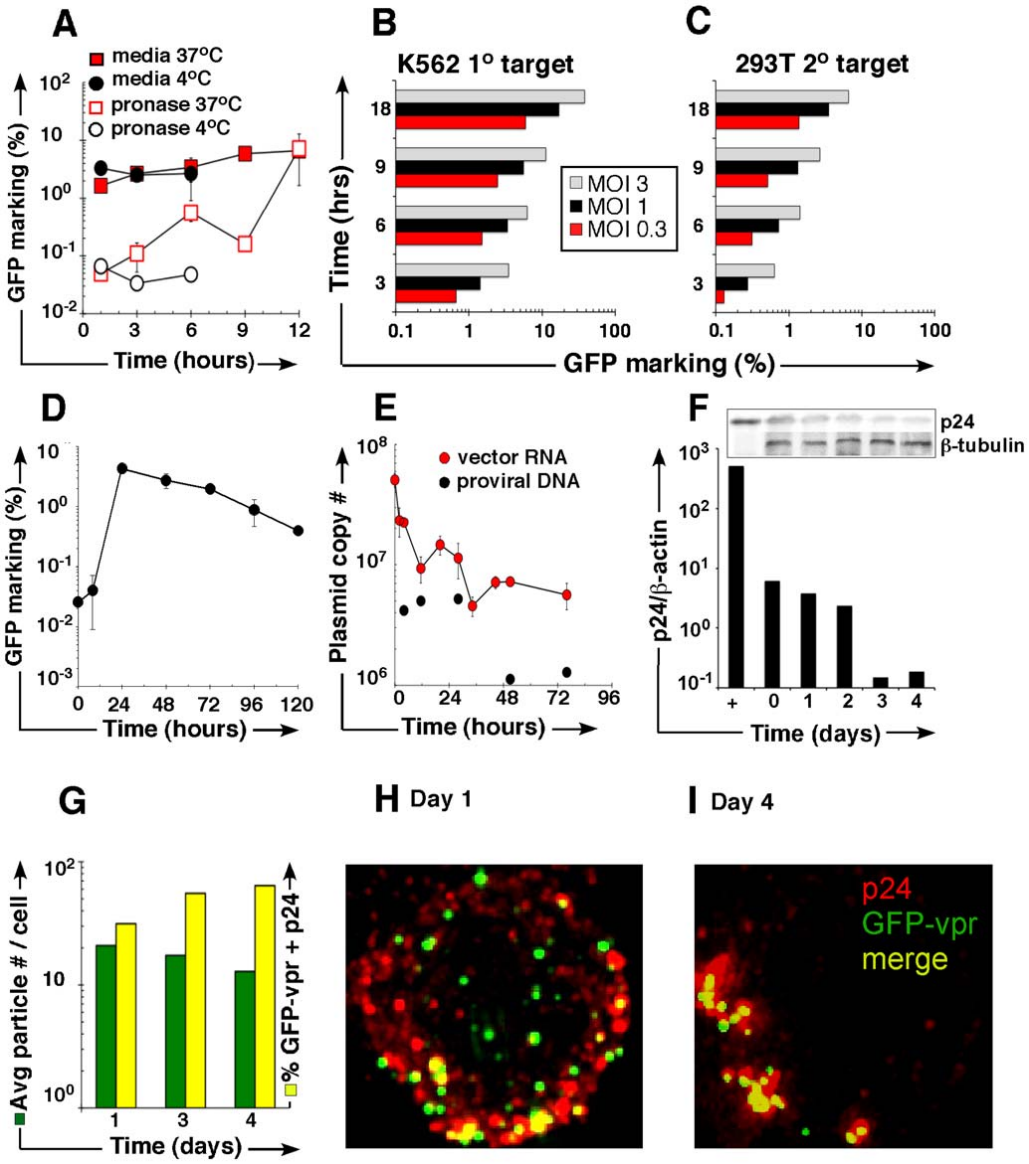
Vector uptake into a protease-resistant compartment and subsequent transfer. (A) GFP marking in (CD45 negative) 293T 2° cells following coculture with vector exposed SupT1 cells washed in media (shaded) or pronase wash (non-shaded). Error bars represent standard deviation between samples. (B) K562 cells were vector-exposed, pronase-washed and co-cultured with 293T cells. CD45-APC staining captured 90% of myeloid population, implying that %GFP positive 293T cells (C) may reflect a minor percentage of admixed residual K562 cells. (D) L1210 cells were exposed to vector at low MOI for 3 hours, followed by pronase wash, independent propagation for designated time (*x-*axis), and coculture with 293T cells. Vector genomes in 293T cells were detected by FACS with exclusion of 1° cells by CD45 staining. (E) L1210 cells were exposed to vector for increasing lengths of time (*x*-axis) and washed with pronase. RNA was collected, reverse transcribed with random hexamer primers, and quantitative real-time PCR was performed using primers to detect proviral GFP sequence (DNA) or dLTR sequence (vector genome RNA). (F) Densiometric analysis and p24 immunoblot (inset). Cell lysates prepared from vector-exposed, pronase-washed Jurkat cells, resolved on a 10% PAGE gel, probed with antibody against p24 or β-tubulin. (G) GFP-vpr labeled genomes (green), anti-p24 was fluorescently labeled AlexaFluor 647 (far-red), and colocalized GFP-vpr with p24 particles (yellow) were counted in each cell. The *y-axis* represents the percent of total cells counted with a given number of colocalized particles. (H, I) Representative images of Jurkat cells on day 1 and 4, respectively, after vector exposure.

We and others previously described the saline wash-resistant persistence of lentivector particles and their conditional transfer to 2° cells [5], [8]. To distinguish prolonged cell surface adherence from intracellular capture, SupT1 cells were exposed to VSV-G pseudotyped GFP-encoding lentivector at 37°C, or 4°C to allow binding while preventing uptake [9], [10], followed by serial washes in PBS and direct co-culture on pre-plated 293T fibroblasts, as indicator cells. GFP expression in (CD45 negative) 293T cells as an indication of genome transfer, integration and expression was seen in both conditions (Fig. 1A). By contrast, vector exposure at 4°C, followed by pronase treatment degraded surface bound particles without significant marking in 293T cells. Remarkably, when vector exposure at 37°C was followed by pronase treatment (at concentrations experimentally determined to degrade surface-bound particles, Fig. S1A) we reliably observed GFP expression in a low (0.1–1%), but consistent percentage of co-cultured 293T cells, progressively increasing in magnitude during extended vector exposure duration in 1° cells (Fig. 1A, open red squares). This is confirmed by real-time PCR studies that show the amplification of proviral GFP sequences in DNA extracted from 293T 2° targets escalating in concert with input MOI during 1° cell exposure (Fig. S1B). Secondary transfer was scalable across a wide range of conditions, and we consistently observed the 1° cell exposure time- and dose-dependent correlation of genome transfer and stable proviral expression in 2° cells (here K562 cells, Fig. 1B,C). Results were confirmed in lymphoid (Raji) cells; HepG2 human hepatoma cells and for alternate (ecotropic) pseudotype and γ-retroviral transfer vectors (Fig. S1C,D). Gain in replication competence (p24Gag ELISA) in vector lots, as well as experimental supernatants in these and subsequent experiments, was specifically excluded. Together, these data reveal that cells retain a fraction of vector genomes in a protease-resistant compartment for transfer to 2° targets.

### Prolonged sequestration and delayed transmission

We next evaluated the rate of genome cell-cell transfer. Murine L1210 cells were exposed to VSV-G GFP vector particles, washed in pronase and independently propagated for up to five days, before transfer to co-culture with 293T cells. Results indicate the transmission of genomes as late as five days after the initial exposure, with a peak at 24 hours, followed by subsequent decline (Fig. 1D). Flow-cytometric detection of GFP expression in 293T cells 16 days after co-culture (293T doubling time: 18 hours, data not shown) exclude simple GFP protein transfer (Fig. S1E). In repeat experiments we collected 1° cell aliquots at serial time points, from 0 to 76 hours after vector exposure for real-time RT-PCR to detect cytoplasmic vector-RNA genome-specific long terminal repeats (LTR). Results confirm that LTR- genomes decline by about 8–10 fold, but remain detectable at all time points examined, at levels specifically determined to exceed residual transcriptional background activity from SIN modified vectors (Fig. 1E) [11], [12]. The resulting curves track the reverse transcription and predicted decay of RNA genomes, as well as the appearance of persisting integrated (DNA) provirus. Additional independent validation comes from the extended detection of p24(Gag) protein by immunoblot and densiometric analysis at serial time points following vector exposure (Fig. 1F). Finally, we used GFP-vpr fusion protein tagged particles and undertook deconvolution immunofluorescent microscopy [13], [14]. HIV-1 derived Vpr binds to the viral capsid (Gag), allowing for cytoplasmic tracking of GFP-vpr tagged particles. Thus, the observed p24 colocalization with GFP-vpr provides further support for the prolonged presence of particle cores at time points up to four days after vector exposure (Fig. 1G–I). Together, multiple lines of evidence lead us to conclude that cells routinely sequester genomes in the cytoplasm where they escape degradation and nuclear translocation.

### Vector cores undergo minimal processing

**Figure 2:**
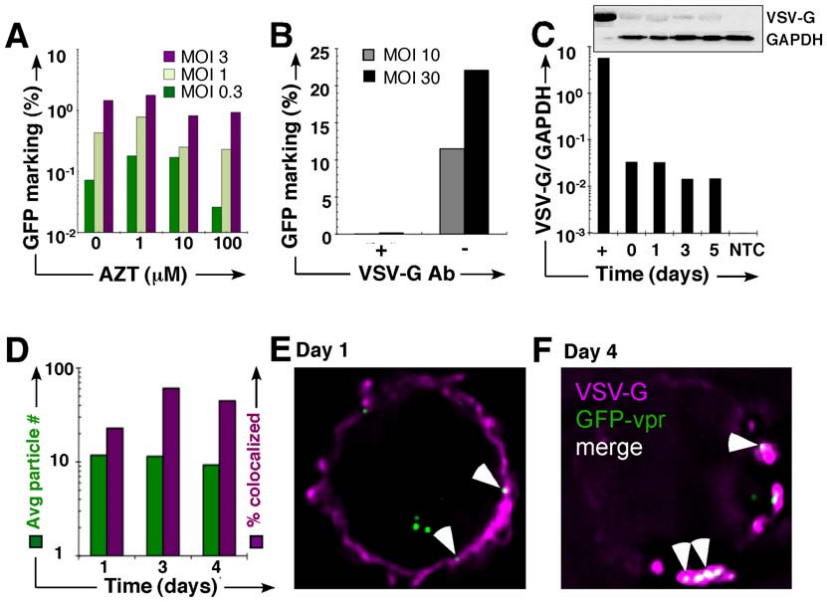
Vector particles persist in an unprocessed state. (A) 293T 1° cells were incubated overnight in AZT, followed by 3-hour vector exposure in the presence of AZT, pronase wash, and co-culture (without AZT) with Jurkat 2° cells. GFP expression was determined in the 2° CD45+ (Jurkat) population by FACS. (B) Jurkat cells were exposed to vector, pronase washed, and placed in co-culture with 293T cells with (+/−) anti-VSV-G neutralizing antibody. GFP was examined by FACS. (C) VSV-G immunoblot of cell lysates prepared from from vector-exposed, pronase-washed Jurkat cells probed with antibody against VSV-G or β-tubulin. The positive control is neat vector; each lane corresponds to the day of (or after) vector exposure. (inset) Densiometric analysis of immunoblot (D) Jurkat cells were exposed to GFP-vpr tagged vector, washed with pronase, and propagated in culture for 4 additional days. The vector genomes are GFP-vpr labeled (green), and anti-VSV-G is fluorescently labeled with the 2° antibody AlexaFluor 647 (far-red). Colocalized GFP-vpr and p24 particles (yellow) were counted in each cell. The left *y-*axis represents the percent of total cells counted with a given number of colocalized particles. The right *y-*axis represents the percentage of particles associated with VSV-G envelope. (E, F) Representative images of Jurkat cell on day 1 and 4, respectively, after vector exposure. GFP-vpr particles (green), anti-VSV-G antiserum (magenta), and colocalized particles (white) are shown.

During the vector life cycle the particle is taken up into the cell, where the core is rapidly uncoated to undergo reverse transcription and generate the pre-integration complex [7]. To test whether reverse transcription itself was limiting to 2( transfer, we pre-incubated 293T cells for 8 hours with increasing concentrations of reverse transcription inhibitor azidothymidine (AZT), followed by a 3-hour vector exposure (+AZT), pronase wash, and direct co-culture with Jurkat cells (- AZT). The almost undiminished levels of genome transfer and GFP expression in 2( cells indicate that reverse transcription is not a requirement for cell-cell transfer (Fig. 2A). To further investigate the extent of genome processing in 1o cells we exposed Jurkat cells to VSV-G pseudotyped vector, followed by pronase wash and direct co-culture with 293T cells, and observed that 2( transfer was almost completely abrogated in the presence of a neutralizing anti-VSV-G antibody, but not IgG isotype (Fig. 2B). This suggests that the vector retains its envelope during cell-cell transfer and that genome processing during cytoplasmic trafficking and transfer from 1( to 2( cell is minimal. Confirming these results, we found no 2( transfer after exposure of murine L1210 cells to ecotropic pseudotyped lentivector and co-culture with (non-permissive) human 293T cells (not shown), indicating that cytoplasmic passage does not appear to alter the tropism for 2( transfer. The detection of VSV-G protein by immunoblot (Fig. 2C) and immunofluorescent deconvolution microscopy (Fig. 2D–F) collected at serial time points following vector exposure further support the sequestration of unprocessed genomes. Concurrent localization of GFP-vpr (*gag*) with VSV-G envelope provides additional validation for the presence of intact particles (Fig. 2D–F). The observed lack of processing following sequestration is consistent with a general cellular pathway rather than conventional vector trafficking.

### Pharmacological modulation of conventional trafficking pathways and 2° transmission

**Figure 3:**
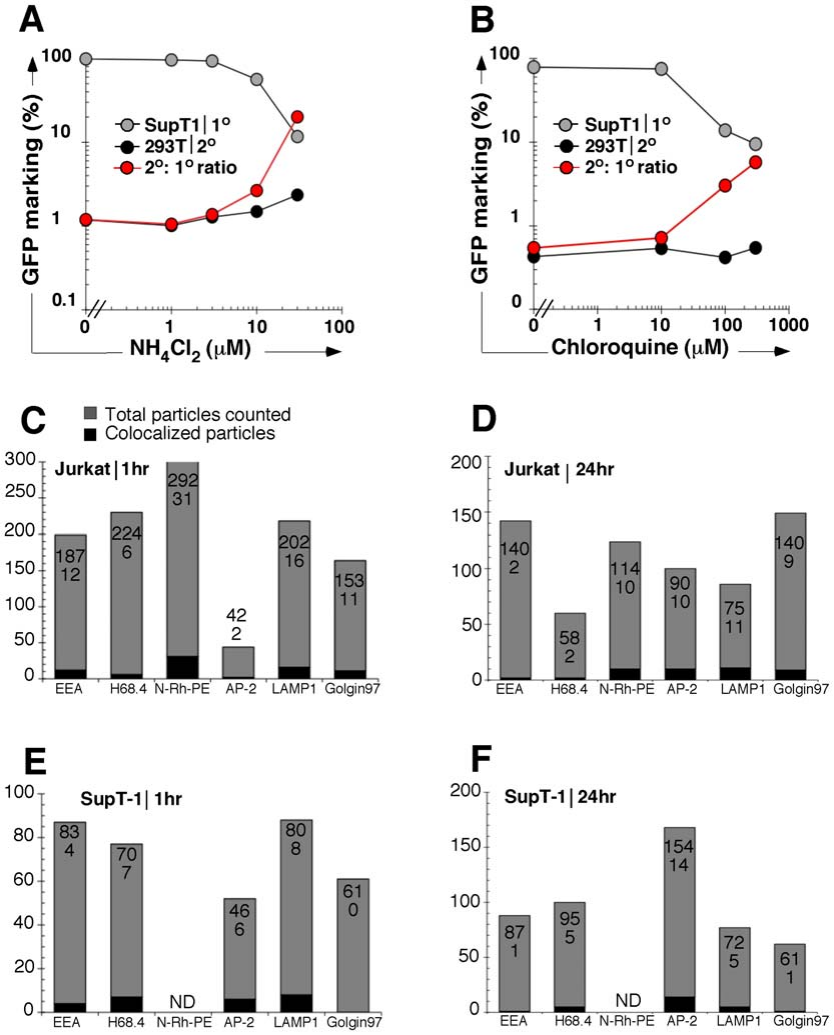
Endosomal acidification increases the proportional efficiency of secondary transfer. (A) Effect of endosomal acidification on 2° transfer. SupT1 carrier cells were pretreated with escalating doses of ammonium chloride or chloroquine (B) followed by vector exposure, pronase wash, and 24-hour coculture with 293T cells. Primary transduction (gray), secondary transfer (black), and proportional % efficiency of secondary transfer (red) are shown. (C,D) Colocalization of vector genomes with representative endosomal markers. Jurkat cells and (E,F) SupT1 cells were exposed to GFP-vpr tagged vector for 1 (C,E) or 24 hours (D,E). Cells were stained with antibodies against indicated endosomal cellular compartments (*x-axis*), as well as Golgin 97 (negative control) and LAMP1 (positive control), and cells were visualized by immunofluorescent microscopy for determination of co-localization of particles and specific compartment markers. The total number of particles (gray, numerator in each column) and total number of co-localized particles (black, denominator in each column) were counted.

Given the pH dependent uptake of VSV-G pseudotyped particles, we next explored the role of endosomal routing by treating SupT1 carrier cells with ammonium chloride, a selective inhibitor of vacuolar H+ ATPases [15]. Treatment was initiated prior to vector exposure and maintained during subsequent co-culture with 293T cells. While we found the anticipated decrease in uptake of particles in carrier cells [15], we noted largely unchanged rates of 2° transfer, in turn resulting in up to a 20-fold proportional increase in the efficiency of 2° transfer to 293T cells (Fig. 3A). These results were confirmed after pH modulation with chloroquine and point to a method for manipulating the trafficking [13] (Fig. 3B). By contrast, when we inhibited proteasome degradation, lysosome transportation, or actin polymerization, we did not observe significant effects on 2° transfer (Fig. S2A–C). The endosome is a principal cellular compartment involved in protein signaling [14], and we performed deconvolution microscopy to determine if it was similarly implicated in trafficking vector genomes. We evaluated the intracellular location of GFP-vpr tagged vector genomes and their colocalization with select endosomal compartments: AP2 (clathrin adaptor protein), EEA1 (early endosomes), H68.4 (transferrin receptor: early and recycling endosomes), and N-Rh-PE (used for tracking endocytosis/exocytosis [16], [17]). Cells were stained with anti-Golgin 97 antibody (Golgi) as a negative control, or an anti-lysosomal antibody (LAMP1) as a positive control for degradation in the lysosome. In both Jurkat and SupT1 cells, up to 10% of genomes were found to colocalize with individual endosomal markers following a 1-hour vector exposure (Fig. 3C,E, Fig. S2C). A similar percentage of particles was found to colocalize with endosomal compartments following a 24-hour vector exposure (Fig. 3D,F, Fig. S2C). Approximately 10% of particles also colocalized with the endosomal/exosomal marker N-Rh-PE in Jurkat cells, at both early and late time points (Fig. 3C,D). Overall, similarly low frequencies of co-localization with Golgi, LAMP1, and endosomal markers illustrate ongoing canonical endosomal trafficking, processing by Golgi, and degradation by the lysosome between the time points examined. We conclude that sequestration and trafficking predominantly occur in a cytoplasmic compartment not identified by conventional endosomal markers.

### Tetraspanin-enriched compartments traffic vector particles in 1° target cells

**Figure 4:**
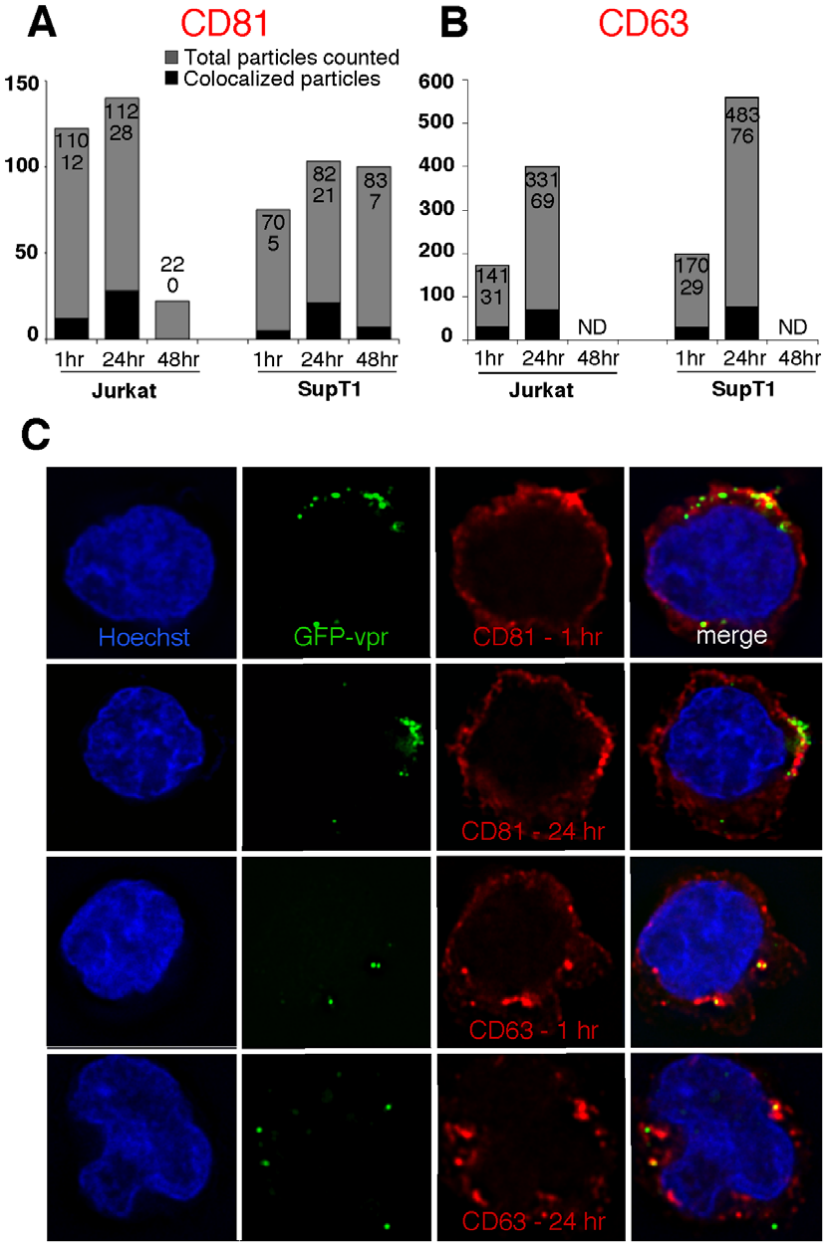
Vector particles are associated with tetraspanin-enriched compartments. Jurkat and SupT1 cells were exposed to vector for 1 or 24 hours. Cells were stained with Hoechst 33342 nuclear stain, washed, fixed, permeabilized, cytospun, and stained with anti-CD81 or anti-CD63 antibody (TAPA1, tetraspanin). (A,B) Vector genome association with select tetraspanins. Columns represent total vector number (gray, numerator in each column) over particles colocalized with tetraspanins (black, denominator in each column). Fluorescent images were deconvolved to confirm intracellular particle location. (C) Representative images illustrating vector genome association with tetraspanins. Particles are GFP-vpr labeled (green), CD81 or CD63 tetraspanin is 2°-labeled with Alexa Fluor 647 (far-red). Yellow indicates colocalization of the two fluorescent signals. Only fully merged and overlapping particles were counted as colocalized.

Tetraspanin proteins associate with endocytic and plasma membranes [18] and organize a number of signaling complexes [19]. To investigate whether sequestration and transmission occur from a tetraspanin-enriched compartment, we serially imaged cells exposed to GFP-vpr tagged vector genomes by deconvolution immunofluorescent microscopy. Results not only demonstrate a cellular distribution of both tetraspanins, CD63 and CD81 consistent with the literature [20], [21], but reveal substantial colocalization of GFP tagged genomes and kinetics that parallel those of 2° transfer observed in co-culture studies (Fig. 1D). Minimal co-localization with CD81 at 1-hour vector exposure is followed by robust (p = 0.002, MANOVA test) gains at 24-hour after vector exposure and a subsequent decline at 48 hours (Fig. 4A,C). By contrast, colocalization of GFP-vpr with CD63 showed relatively little variation over time (Fig. 4B,C).

**Figure 5:**
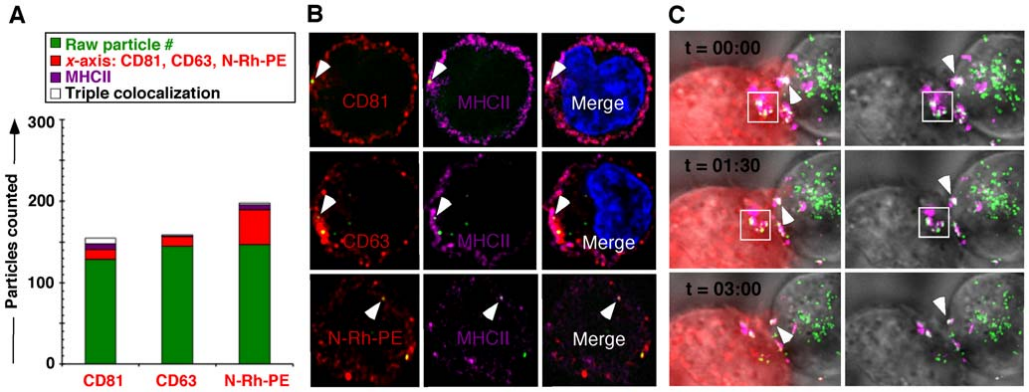
Vector particles associate with MVB markers. (A,B) Vector genomes associate with select MVB markers. Jurkat cells exposed to GFP-vpr vector (green) overnight, followed by pronase wash, and stain with antibodies against CD81, CD63, or N-Rh-PE (red), MHCII (magenta). Particles found associated with CD81 and MHCII, or N-Rh-PE and MHCII are white. (C) Live cell imaging of vector-exposed, pronase-washed 1° SupT1 cells (labeled with anti-CD81, Alexa Fluor 647, magenta) in co-culture with 2° 293T DsRed actin (red) expressing cells. Right hand panels lack the DsRed layer for improved visual clarity of otherwise identical frames. Genomes co-localized with tetraspanin are white (arrows, boxes). Genomes co-localized with DsRed actin are yellow. Deconvolution microscopy was performed on live cells by collecting series of *z*-stacks (0.5 µm) every minute for 10 minutes, elapsed time is indicated in black.

Tetraspanin proteins are constitutive components of exosomes, which derive from structures termed multivesicular bodies (MVB) that are involved in cellular trafficking [22]. Reflecting the potential involvement of this organelle in sorting material to exosomes [23] we investigated its role in trafficking genomes. These imaging studies revealed colocalization of GFP-vpr labeled genomes and tetraspanins with MHC class II protein, and the exocytosis marker N-Rh-PE [24], supporting the role of MVB in sequestration and cell-cell transfer (Fig. 5A,B). Looking to provide additional support for the transfer of genomes among 1° (SupT-1) and 2° target cells (293T, distinguished by their DsRed labeled actin cytoskeleton) we performed real-time deconvolution microscopy to directly track genomes trafficked in MVBs (i.e. CD81 associated). We labeled 1° cells with anti-CD81 (magenta) and tracked GFP-vpr tagged genomes (green) during transfer. Early on during imaging, CD81 colocalized particles (magenta/green overlay = white, arrow) cluster at the limiting membrane. Successfully transferred genomes then colocalize with DsRed actin (magenta/green/red overlay = yellow, box) inside the 2° cells (Fig. 5C).

**Figure 6:**
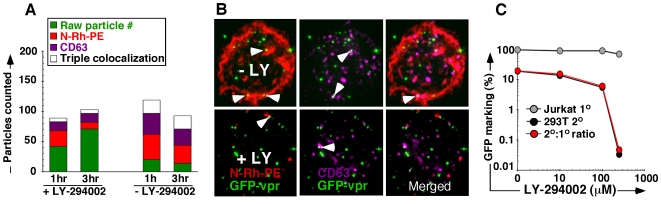
Inhibiting MVB formation abrogates 2° transfer. (A) Enumeration of GFP-vpr vector genomes (green) associated with select MVB markers (N-Rh-PE, red and CD63 tetraspanin, magenta) following exposure to 100 µM LY-294002. Cell aliquots were collected at 1 and 3 hours following exposure. (B) Top panels are representative images from untreated cells, bottom panels representative of treated cells. (C) Functional effect of LY-294002 on 2° transfer of vector genomes. Jurkat carrier cells were pretreated with escalating doses of LY-294002 as in (A,B) followed by vector exposure for 3 hours in the presence of the inhibitor, pronase wash, and 24 hour co-culture with 293T cells. Primary marking (gray), 2° transfer (black), and % efficiency of 2° transfer (red) are shown.

MVB formation can be experimentally disrupted by specific inhibition of phosphatidyl inositol 3-kinase (PI3-K) with LY-294002 [24]. We wished to directly visualize the effect MVB disruption had on genome trafficking in vector-exposed Jurkat cells and concurrently exposed cells to LY-294002, the exocytosis marker N-Rh-PE, and GFP-vpr vector, followed by pronase wash and stain with anti-CD63. While colocalization with CD63 remained relatively unaffected, association with N-Rh-PE decreased with time, in contrast to the non-treated control (Fig. 6A, B). To confirm the functional relevance of MVB involvement, we repeated this experimental set-up in a co-culture assay and observed a LY-294002 dose-dependent decrease in 2° transfer (Fig. 6C). Along with co-culture transduction studies, these observations indicate that blocking MVB formation results in altered cytoplasmic trafficking and subsequent decrease in 2° transfer of genomes. In sum, these experiments implicate MVB in sequestration and 2° transfer of RNA-genomes.

## Discussion

The physiologic transfer of information between eukaryotic cells relies on protein-based signaling cascades. More recent studies have reported the horizontal, non-infectious, transfer of genetic sequence (RNA) by MVB-derived exosomes and microvesicles between highly specialized cells [2], [3], [4], [25], [26]. Those reports lend general support to a model of horizontal RNA transfer and genetic cell-cell communication, but focus on the “passive” detection of microvesicles/exosomes released from immune and other specialized cells with the demonstration of rare and transient donor cell derived biologic effects in 2° target cells [27], [28]. To better understand the nature and therapeutic potential of such a process, we chose an alternate approach that allowed us to shift the experimental focus to trafficking in 1° cells, and the demonstration of heritable effects in 2° target cells.

We previously reported the cell-cell transfer of VSV-G pseudotyped, replication-incompetent HIV-1 derived vector from hematopoietic cells to 2° targets [5]. Systematic dissection of this observation using transduction rescue assays, PCR analyses for vector genomes, western blotting and deconvolution microscopy, studies herein provide compelling evidence that a wide range of cell types are capable of successfully transmitting vector genomes in a process that is scalable (dose-dependent) at the level of vector input. Sequestered, GFP-vpr tagged genomes were found to localize at the cell membrane of the 1° cell up to four days after exposure (last time point tested). Indeed, the delayed visualization of GFP-vpr labeled (i.e. Gag associated) genomes in 1° and 2° target cells by deconvolution microscopy indicates that this does not simply represent horizontal transfer of circular LTR-DNA species, for which nuclear processing and loss of Gag are prerequisites [29]. Moreover, the delayed detection of provirus by quantitative real-time PCR analysis in rapidly dividing 2° targets demonstrates stable integration and excludes the mere transfer of protein products [29], [30]. In fact, undiminished cell-cell transfer after RT inhibition in 1° vector-exposed cells implies that genomes do not complete core processing, nor is there a requirement for integration into the primary target.

**Figure 7:**
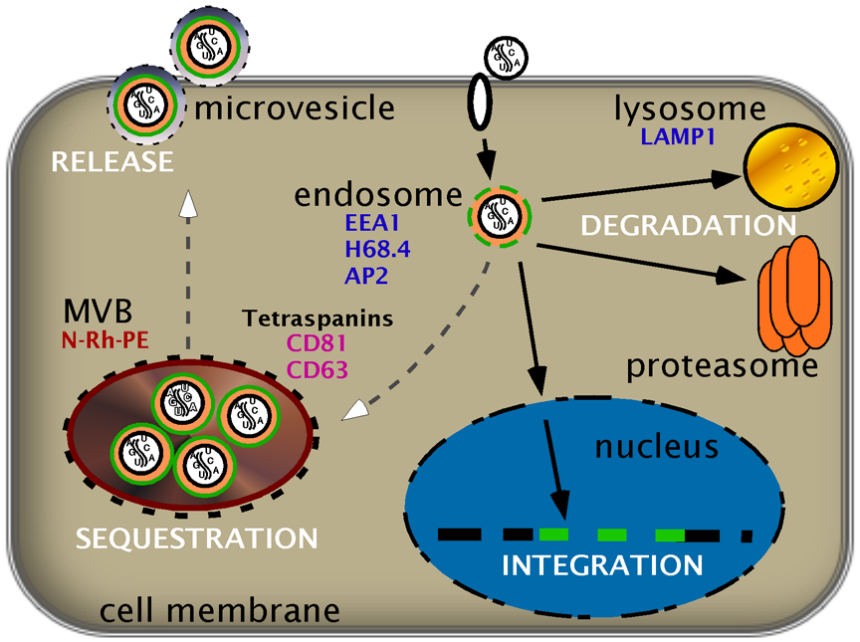
Working model of intracellular trafficking and horizontal transfer of RNA genomes. Particles carrying RNA genomes are taken up into a cell, processed, and traffic the cytoplasm destined for nuclear integration or degradation by the proteasome or lysosome. Alternatively, some genomes are retained in MVB (*kathexis*), avoid processing, and can be released to transfer to 2° cells. Cell markers (e.g. antibodies) used in these experiments are listed below their corresponding compartment.

Because endosomes are involved in protein-mediated cell signaling [14], and VSV-G pseudotyped particles enter the cell through endocytosis, we initially tested involvement of the endosomal compartment, but found only low-level constitutive colocalization with endosomal markers (EEA1, transferrin receptor, clathrin adaptor AP-2) by immunofluoresecent microscopy. On the other hand, we observed significant intracellular colocalization of GFP tagged genomes with tetraspanins, CD63 and CD81, that peaks at 24-hours, in kinetics reflecting our functional studies. In distinct contrast to viral trafficking [22], 2° transfer capacity was unaffected by experimental disruption of canonical processing involving proteasomal and lysosomal function, or depolymerization of the actin cytoskeleton [4], [25], [31], [32]. Intriguingly, these attributes are consistent with microvesicle involvement, whereby multivesicular bodies (MVB) evolve from late endosomes to overcome the subcortical actin barrier, and fuse with the plasma membrane to release their exosome content [33], [34], [35]. In keeping with this hypothesis, we observed significant colocalization of genomes with proteins enriched in MVB, including the exocytosis marker (N-Rh-PE) and, albeit to a lesser extent, MHC II [20], [28] in kinetics again corresponding with functional assays of 2° transfer. Indeed, specific inhibition of MVB formation with LY-294002 resulted in time-dependent loss of genome colocalization with N-Rh-PE in imaging experiments as well as dose-dependent diminution of 2° transfer in co-culture experiments. Collectively, these experiments support the involvement of MVB structures in particle retention (*kathexis*), and the directed transfer of genetic material between cells. Unlike existing studies [22], [27], [36], these results imply a horizontal RNA transfer pathway between cells that is accessible to integration competent genomes, and therefore directly amenable to manipulation, Fig. 7. Two alternate, not mutually exclusive, hypotheses may explain what directs genomes to this pathway. Work by Skog and colleagues suggests that specific RNA sequence tags may be responsible for directing genomes into an exosomal pathway [2]. This possibility is consistent with the detection of endogenous retroviral RNA species in exosomal preparations [37]. Alternatively, gag itself may be responsible for sorting cores into microvesicle export pathways [20], [38]. In either case, the transfer of genetic information allows for prompt adaptation of cell populations to alterations in the microenvironment, and may help explain why hypoxia, irradiation, or cell activation augment microvesicle release [2].

While reported instances of exosomal transfer have demonstrated endogenous cellular protein, and more recently RNA, as microvesicular cargo [39], data presented here for the first time suggest that such a transfer pathway can be actively targeted for delivery of replication deficient particles. Thus, at the level of the 2° target tissue, both stable sequence over-expression as well as RNAi based target ablation can be conceptually accommodated and specific molecular targets can be pursued. Our findings imply a novel therapeutic approach for the selective and scalable delivery of genetic sequence in a desired tissue- and cell-specific manner.

## Materials and Methods

### Cell culture

SupT1 cells (a human T-cell line) were grown in RPMI (Gibco) supplemented with 10% FBS (Gibco), 1% Penicillin/Streptomycin (Pen/Strep, Gibco), 1 mM sodium pyruvate, 10 mM Hepes, and 2 mM L-glutamine. HepG2 human hepatoma cells were grown in MEM (Gibco) supplemented with 10% FBS, 1% Pen/Strep, 1 mM sodium pyruvate, and 1 mM nonessential amino acids (Gibco). HEK 293T human kidney fibroblasts and NIH 3T3 murine fibroblasts were grown in DMEM (Gibco) supplemented with 10% FBS and 1% Pen/Strep. Human Jurkat T-cells, murine lymphoid L1210 cells, and human K562 myeloid leukemia cells were all grown in RPMI supplemented with 10% FBS and 1% Pen/Strep.

For direct co-culture experiments, 1×10^5^ 293T cells were seeded in a 12-well tissue culture plate (Costar). Vector-exposed non-adherent hematopoietic cells were washed and placed alongside pre-plated fibroblasts for 24 hours, after which time non-adherent cells were aspirated. Residual cells were stained with anti-CD45 and analyzed by flow-cytometry to distinguish hematopoietic cells from fibroblasts (below). For pronase washes, cells were pelleted immediately following transduction and resuspended in 1 mg/ml pronase (Roche) for 10 minutes at 37°C followed by two washes in media containing 10% FBS. Cells were then re-suspended in corresponding culture media.

In select experiments we pretreated 1° cells with MG 132 (EMD), Bafilomycin A (PKC Pharmaceuticals), Ammonium Chloride (Fisher), Chloroquine (Sigma), AZT (Sigma), Latrunculin A and B (Biomol). In specified experiments these reagents were also present during co-culture. The VSV-G neutralizing antibody was collected from the supernatant of a hybridoma (CRL-2700, American Type Culture Collection) and concentrated with Amicon Ultra columns (Millipore). Anti-IgG antibody was generously provided by Philip Streeter, OHSU Stem Cell Center.

### Vector preparation and transduction

Human 293T kidney fibroblasts cells were seeded at a density of 1.6×10^7^ cells per 15-cm tissue culture dish (Corning), precoated with 0.01% poly-L-lysine (Sigma). The lentivirus transfer vector pRRL SIN EF1α cPPT EGFP wpre LoxP (pWPXL-EGFP) was kindly provided by D. Trono (Geneva, Switzerland). The GFP-vpr plasmid was provided by Eric Barklis (Portland, OR). The γ-retrovirus vector pMND MFG eGFP was kindly provided by Donald Kohn. Calcium phosphate transfection of lentivector packaging (pMD-Lg/p-RRE, pRSV-Rev) and VSV-G envelope plasmids was performed in DMEM, 10% FBS, 1% Pen/Strep. Vector supernatant was harvested 36, 48, and 72 hours later, filtered through a 0.45 µm filter, ultra-concentrated over 30 hours at 7300 RCF, and the pellet was resuspended in Iscove's media (Gibco) and stored at -86°C until use. Limiting dilution titers were determined by FACS and calculated using 293T cells, as previously described [40]. Cells were washed and resuspended in corresponding media (described below), with 4 µg/ml protamine sulfate (MP Biomedicals). Transductions took place at 37°C or 4°C for specified lengths of time.

### Flow-cytometry

Retroviral transduction was analyzed by GFP expression using a FACS-Calibur instrument (BD Biosciences) using Flow Jo software (Tree Star, Ashland, OR). For determination of cytoplasmically located vector genomes, cells were collected and analyzed following vector exposure; for determination of events that resulted in nuclear integration, cells were collected and analyzed 72 hours following vector exposure (to allow for transcription, translation, and processing of protein). At least one hundred thousand events were collected for any given sample. Cells of hematopoietic lineage were determined by staining with anti-CD45 (murine, PE-conjugated, BD Biosciences or human, APC conjugated, eBioscience). Non-viable cells were excluded from analysis by uptake of propidium iodide (1 µg/ml).

### p24 Enzyme-linked immunosorbent assay (ELISA)

To verify replication incompetence, testing was performed by transducing SupT1 cells with vector supernatant for 24 hours, followed by washing, and serial passage of cells for 2 weeks in culture. Cellular supernatant was collected for p24 (Gag) ELISA (Perkin-Elmer, Boston, MA) and samples were run in duplicate per manufacturer's protocol. No samples tested positive.

### Quantitative real time RT-PCR

Total RNA was extracted from cells using an RNeasy Mini Kit, according to manufacturer's protocol (Qiagen Inc., Valencia, CA) and subjected to DNase treatment. Complementary DNA was made using SuperScript™ RT (Invitrogen). Expression was determined via quantitative real-time PCR on a StepOne Plus ABI sequence detection system (Applied Biosystems). The primers used to detect proviral GFP were sense: 5′ GTG GTG CCC ATC CTG GTC GAG C -3′ and anti-sense: 5′- CAC CAG GGT GTC GCC CTC GAA C -3′. The primers used to detect proviral long terminal repeats (dLTR) were sense: 5′-TGT GTG CCC GTC TGT TGT GT-3′ and anti-sense: 5′-GAG TCC TGC GTC GAG AGA GC-3′. Amplification using random hexamer or oligo dT primers exhibited similar kinetics, providing internal validation. The primers used to detect the endogenous GapDH endogenous control were sense: 5′- AAA TAT GAC AAC TCA CTC AAG ATT GTC A -3′ and anti-sense: 5′- CCC TTC CAC AAT GCC AAA GT -3′. Reactions were set up in a MicroAmp Optical 96-well Reaction Plate (Applied Biosystems), amplified using Power SYBR Green PCR Mastermix (Applied Biosystems), and run in triplicate. All threshold cycle (Ct) values of GFP were normalized to GapDH endogenous control Ct values. For determination of plasmid copy number, dilutions of plasmid containing dLTR and GFP were titrated and quantitative real-time PCR was performed using each primer set. A standard linear regression model was applied to determine the best fit between lines to compensate for differences in amplification efficiency (for variance between intercepts and slope p<0.001).

### Deconvolution Microscopy

Deconvolution microscopy was performed at the OHSU Department of Molecular Microbiology and Immunology Shared Resource. The Applied Precision Deltavision Image Restoration System™ includes a chassis with precision nano-motorized XYZ stage, an Olympus IX71 wide field microscope, a Nikon Coolpix HQ Camera; and DeltaVision SoftWoRx™software. Deconvolution is performed with SoftWoRx software (Applied Precision), and additional image processing is performed with Bitplane Imaris™software. Images were acquired using the 60× 1.4NA oil lens. Z-stacks of 3 colors (Hoechst33342, GFP, and Alexa-Fluor 647) were acquired at 0.5 µm for the complete depth of the cells (approximately 19–20 Z-planes) and were deconvolved for 9 iterations with the appropriate (experimentally determined) point spread function (PSF). Histograms were adjusted to display the data as 24 bit RGB tiffs and movies. Rotational movies were made from 3D volumes created in Imaris (ver. 5.7.2). Adobe Photoshop was used to separate color channels.

### Immunofluorescence

Jurkat and SupT1 cells were transduced with GFP expressing vector particles (GFP-vpr) at a density of 2.5×10^5^ cells for designated transduction times, followed by Hoechst 33342 staining (5 µg/ml, Invitrogen) for 1 hour. Cells were fixed for 15 minutes at room temperature in 4% paraformaldehyde, and permeabilized for 10 minutes at 4°C with NET buffer (150 mM NaCl, 5 mM EDTA, 10 mM Tris, pH 7.4, with 0.5% triton X-100), as previously described [41]. Cells were then cytospun onto glass slides. Cells were stained for 1 hour with 1° antibody in PBS with 2% FBS (Gibco), washed, stained for 1 hour with 2° antibody (Alexa Fluor 647, Invitrogen) in PBS with 2% FBS, washed, and mounted with Fluoromount G (Fisher) onto glass slides. The 1° antibodies used were anti-CD63 (BD Biosciences), anti-CD81 (Abcam), anti-AP2 (Abcam), anti-MHCII (Abcam) and anti-transferrin receptor (H68.4, Zymed). EEA1 and Golgin 97-specific antibodies were generous gifts from Dr. Caroline Enns (Portland, OR). The monoclonal antibodies against LAMP-1 and CD63 were developed by J. Thomas August and James E.K. Hildreth, and were obtained from the Developmental Studies Hybridoma Bank developed under the auspices of the NICHD and maintained by The University of Iowa, Dept. of Biological Sciences, Iowa City, IA 52242. The p24 antiserum was collected from the supernatant of murine Hy183 hybridoma cells, generously provided by Dr. Eric Barklis. The number of cells examined in the experiments illustrated in each figure is given in Table S1.

To determine extended duration of genomes, Jurkat cells were exposed to GFP-vpr tagged vector, washed with pronase, and propagated in culture for 4 additional days. Cell aliquots were prepared for imaging (cytospun and stained with corresponding antibodies) serially every 24 hours.

To trace endocytic/exocytic pathways, Jurkat and SupT1 cells were exposed to 5 µg/ml Lissamine rhodamine B (N-Rh-PE, Avanti Polar Lipids, Alabaster, AL) for 1 hour (endocytosis) or 24 hours (exocytosis). Cells were washed in PBS and prepared, as described, for microscopy.

To visualize the effect of PI3-K inhibition, 2.5×10^5^ Jurkat cells were pretreated for 30 min with LY-294002 (EMD Bioscience), followed by a 3-hour vector exposure in the presence of the inhibitor, pronase wash, and preparation for imaging as described above.

For visualization of actin filaments, 293T were stably transfected with DsRed-Monomer-Actin Vector (Clontech). DsRed-expressing cells were single-cell sorted on an Influx instrument (Cytopeia) and cultured in 2 mg/ml G418 (Invitrogen) for isolation and maintenance of stably integrated clonal events.

### Western Blotting

Cells were exposed to VSV-G pseudotyped vector (MOI 3) for 5 hours, followed by pronase wash. At serial time points, 1.5×10^6^ cells were pelleted and lysed with buffer containing 0.15 M NaCl, 5 mM EDTA, 1% Triton X-100, 10 mM Tris-Cl, Halt Protease Inhibitor Cocktail (Thermo Scientific), and 5 mM DTT. Lysates were resolved on a 10% polyacrylamide gel, transferred to membrane, stained with 1° antibody p24 anti-serum, VSV-G antibody (Sigma), or antibody against β-tubulin (Santa Cruz Biotechnologies) or GAPDH, and anti-HRP 2° antibody (Amersham). Images were visualized on a Lumi-Imager (Roche Applied Science) and densiometric analysis was performed with LumiAnalyst 3.1 software.

### Statistics

Numerical results are expressed as average plus or minus standard deviation (SD). Data were analyzed using the paired 2-tailed Student t-test. P values of less than 0.05 were considered significant. For determination of significance of co-localization in microscopy experiments, a multivariate analysis of variance (MANOVA) was performed. To confirm results, an F-test was performed.

## Supporting Information

Figure S1 (A) Vector was incubated in increasing concentrations of pronase for 10 minutes, followed by vector exposure in murine L1210 cells. GFP marking was determined by FACS. (B) Vector genomes detected in 293T cell DNA 72 hours post-coculture with vector-exposed, pronase-washed L1210 cells by qRT-PCR with GFP-specific primers. (C) Raji (human B) cells were exposed to increasing numbers of vector genomes overnight, followed by pronase wash and coculture with 293T cells. GFP marking is shown in 1o Raji (closed circles) and 2o 293T cells (open circles). (D) Murine L1210 hematopoietic cells were exposed to increasing numbers of γ−oncoretroviral vector genomes overnight, followed by pronase wash and coculture with 293T cells. GFP marking is shown in 1o L1210 (closed circles) and 2o 293T cells (open circles). (E) Representative FACS plots generated from samples of 293T 2o cells corresponding to (Fig. 1D) analyzed 16 days following completion of co-culture. Time points indicate the delay between vector exposure of 1o cells and initiation of co-culture (0-, 24-, 48-, 72 hr)
(0.56 MB TIF)

Figure S2 (A) Effect of inhibition of canonical viral trafficking pathways on 2o transfer. Jurkat carrier cells were pretreated with escalating doses of each inhibitor, followed by vector exposure, pronase wash, and 24-hour coculture with 293T cells. GFP marking in 2o cells is shown. Trafficking pathways targeted are: proteosome (MG 132), lysosome (Bafilomycin A), actin-cytoskeleton (Latrunculin B). To confirm that doses of Latrunculin B used had biologic effect on the cells, the experiments were repeated with Latrunculin A, with similar results observed (not shown). Therefore, GFP-vpr vector-exposed cells were treated with 1 µM Latrunculin A, cells were stained with anti-CD63 (far-red). (B) Genomes (green) associated with CD63 were enumerated in cells without (top panels) or following (bottom panels) Latrunculin A treatment. (C) The difference in genomes associated with CD63 following Latrunculin A treatment was statistically different from non-treated control, confirming that the doses of inhibitor used exerted a biologic effect on the cells. (D) Representative images of vector genomes colocalized with endosomal markers in Jurkat cells following a 1-hr or 24-hr exposure (from Fig. 3C,D).
(2.86 MB TIF)

Table S1 Number of cells counted in the experiments used to generate figure panels.
(0.07 MB RTF)
