# Supplementary figures and images for: Cellular Microvesicle Pathways Can Be Targeted to Transfer Genetic Information between Non-Immune Cells
Source: PLoS One. 2009 Jul 13;4(7):e6219. doi: 10.1371/journal.pone.0006219 (PMC2704871; doi:10.1371/journal.pone.0006219)

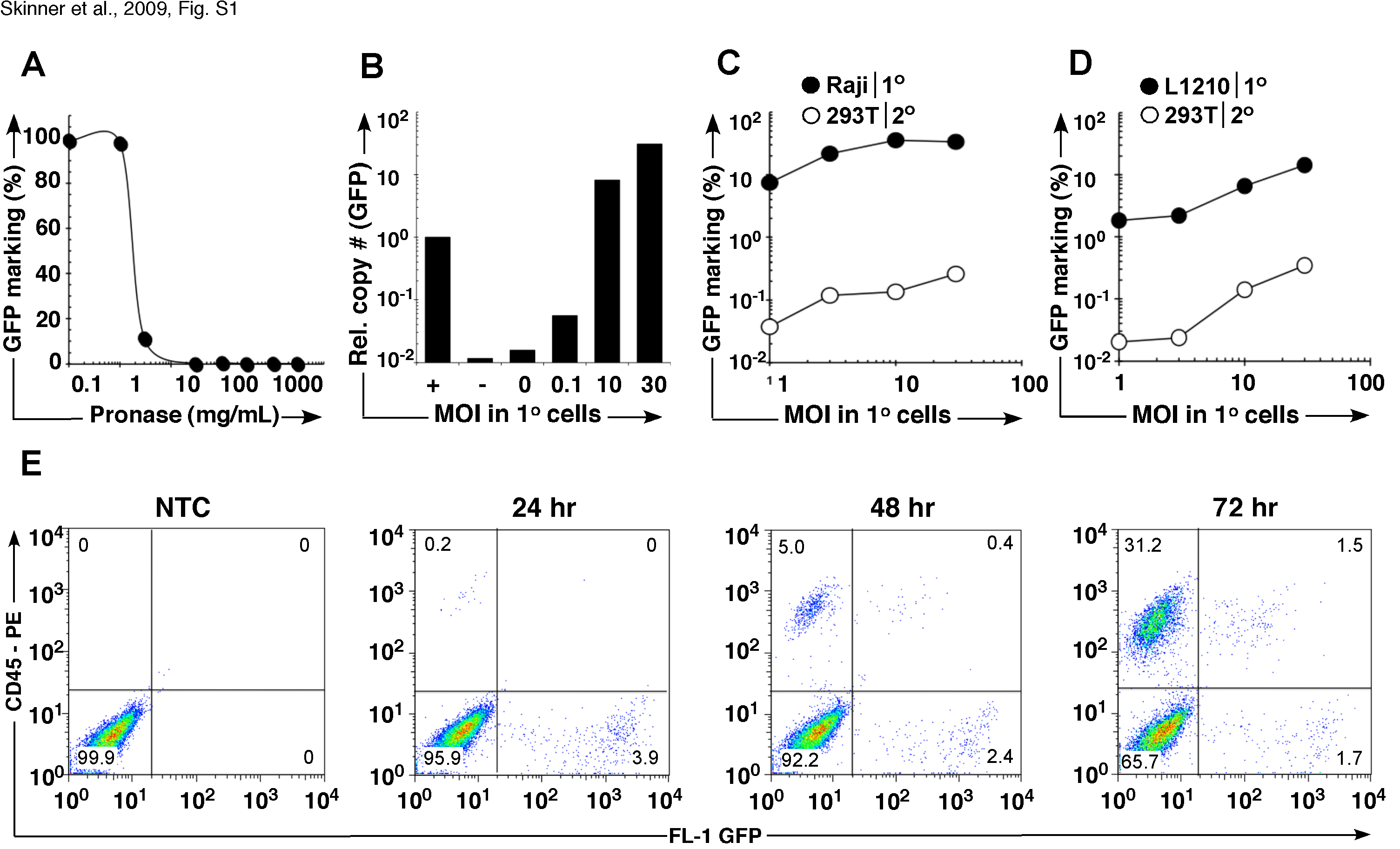

Supplement: Figure S1 — (A) Vector was incubated in increasing concentrations of pronase for 10 minutes, followed by vector exposure in murine L1210 cells. GFP marking was determined by FACS. (B) Vector genomes detected in 293T cell DNA 72 hours post-coculture with vector-exposed, pronase-washed L1210 cells by qRT-PCR with GFP-specific primers. (C) Raji (human B) cells were exposed to increasing numbers of vector genomes overnight, followed by pronase wash and coculture with 293T cells. GFP marking is shown in 1o Raji (closed circles) and 2o 293T cells (open circles). (D) Murine L1210 hematopoietic cells were exposed to increasing numbers of γ−oncoretroviral vector genomes overnight, followed by pronase wash and coculture with 293T cells. GFP marking is shown in 1o L1210 (closed circles) and 2o 293T cells (open circles). (E) Representative FACS plots generated from samples of 293T 2o cells corresponding to (Fig. 1D) analyzed 16 days following completion of co-culture. Time points indicate the delay between vector exposure of 1o cells and initiation of co-culture (0-, 24-, 48-, 72 hr) (0.56 MB TIF) [file pone.0006219.s001.tif]

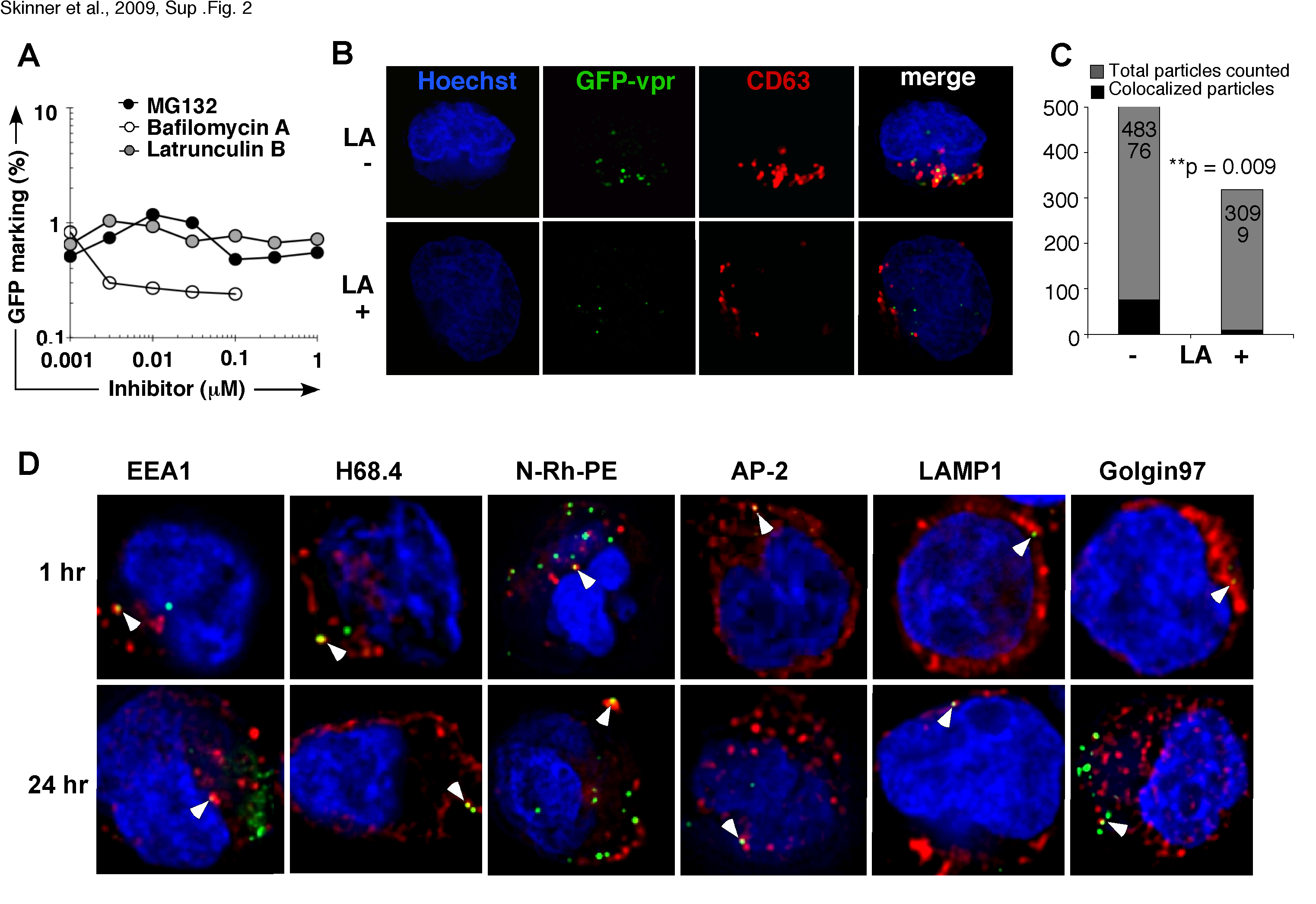

Supplement: Figure S2 — (A) Effect of inhibition of canonical viral trafficking pathways on 2o transfer. Jurkat carrier cells were pretreated with escalating doses of each inhibitor, followed by vector exposure, pronase wash, and 24-hour coculture with 293T cells. GFP marking in 2o cells is shown. Trafficking pathways targeted are: proteosome (MG 132), lysosome (Bafilomycin A), actin-cytoskeleton (Latrunculin B). To confirm that doses of Latrunculin B used had biologic effect on the cells, the experiments were repeated with Latrunculin A, with similar results observed (not shown). Therefore, GFP-vpr vector-exposed cells were treated with 1 µM Latrunculin A, cells were stained with anti-CD63 (far-red). (B) Genomes (green) associated with CD63 were enumerated in cells without (top panels) or following (bottom panels) Latrunculin A treatment. (C) The difference in genomes associated with CD63 following Latrunculin A treatment was statistically different from non-treated control, confirming that the doses of inhibitor used exerted a biologic effect on the cells. (D) Representative images of vector genomes colocalized with endosomal markers in Jurkat cells following a 1-hr or 24-hr exposure (from Fig. 3C,D). (2.86 MB TIF) [file pone.0006219.s002.tif]
