# Supplementary material for: Cellular Microvesicle Pathways Can Be Targeted to Transfer Genetic Information between Non-Immune Cells
Source: PLoS One. 2009 Jul 13;4(7):e6219. doi: 10.1371/journal.pone.0006219 (PMC2704871; doi:10.1371/journal.pone.0006219)
Supplement: Table S1 — Number of cells counted in the experiments used to generate figure panels. (0.07 MB RTF) [file pone.0006219.s003.rtf]

n=number of cells counted
Figure 1	H	I	
	n=20	n=42	
Figure 2	F	G	
	n=22	n=37	
Figure 3	C	D	E	F	
EEA1
H68.4
N-Rh-PE
AP-2
LAMP1
Golgin 97	n=39
n=37
n=33
n=22
n=33
n=41	n=10
n=14
n=23
n=12
n=12
n=15	n=18
n=14
ND
n=30
n=12
n=11	n=7
n=10
ND
n=11
n=9
n=11	
Figure 4	A	B	
Jurkat 1hr
Jurkat 24 hr
Jurkat 48 hr
SupT1 1 hr
SupT1 24 hr
SupT1 48 hr	n=10
n=13
n=7
n=10
n=8
n=10	n=25
n=28
ND
n=25
n=34
ND	
Figure 5	A	
CD81
CD63
N-Rh-PE	n=30
n=20
n=11	
Figure 6	A	
+LY294002 1hr
+LY294002 3hr
-LY294002 1hr
-LY294002 3hr	n=15
n=12
n=8
n=11	
Fig. S2	C	
-LA
+LA	n=11
n=20	
